# Novel prognostic prediction model constructed through machine learning on the basis of methylation-driven genes in kidney renal clear cell carcinoma

**DOI:** 10.1042/BSR20201604

**Published:** 2020-07-21

**Authors:** Weihao Tang, Yiling Cao, Xiaoke Ma

**Affiliations:** 1 Chengdu Foreign Language School, International Department, Class AP-1, Grade 2019, Chengdu, Sichuan, China; 2 West China School of Medicine, Sichuan University, Chengdu, Sichuan, China; 3 School of Computer Science and Technology, Xidian University, Xi’an, Shaanxi, China

**Keywords:** KIRC, methylation-driven gene, methylation, prognostic prediction model

## Abstract

Kidney renal clear cell carcinoma (KIRC) is a common tumor with poor prognosis and is closely related to many aberrant gene expressions. DNA methylation is an important epigenetic modification mechanism and a novel research target. Thus, exploring the relationship between methylation-driven genes and KIRC prognosis is important. The methylation profile, methylation-driven genes, and methylation characteristics in KIRC was revealed through the integration of KIRC methylation, RNA-seq, and clinical information data from The Cancer Genome Atlas. The Lasso regression was used to establish a prognosis model on the basis of methylation-driven genes. Then, a trans-omics prognostic nomogram was constructed and evaluated by combining clinical information and methylated prognosis model. A total of 242 methylation-driven genes were identified. The Gene Ontology terms of these methylation-driven genes mainly clustered in the activation, adhesion, and proliferation of immune cells. The methylation prognosis prediction model that was established using the Lasso regression included four genes in the methylation data, namely, *FOXI2, USP44, EVI2A*, and *TRIP13*. The areas under the receiver operating characteristic curve of 1-, 3-, and 5-year survival rates were 0.810, 0.824, and 0.799, respectively, in the training group and 0.794, 0.752, and 0.731, respectively, in the testing group. An easy trans-omics nomogram was successfully established. The C-indices of the nomogram in the training and the testing groups were 0.8015 and 0.8389, respectively. The present study revealed the overall perspective of methylation-driven genes in KIRC and can help in the evaluation of the prognosis of KIRC patients and provide new clues for further study.

## Introduction

Kidney renal clear cell carcinoma (KIRC) is the most common type of renal carcinoma and accounts for approximately 80–90% of all kidney tumors [1]. KIRC is characterized by a high risk of metastasis and the main cause of death from kidney tumors [2,3]. With the application of medical imaging, such as computed tomography and ultrasound, the early discovery of tumor is improved. However, despite substantial advances in its diagnosis and treatment, the mechanisms underlying KIRC are not fully understood, and the prognosis is still poor [4].

DNA methylation is an important epigenetic modification to regulate gene expression. In some promoter-related cytosine–phosphate–guanine (CpG) sites, DNA methylation often contributes to the silencing of downstream genomic regions [1]. Studies show that tumor suppressor genes can be inhibited by hypermethylation, whereas oncogenes can be activated by hypomethylation [5,6]. Many studies have explored the abnormal DNA methylation in cervical [6] and breast [7] cancers, which may be possible markers for diagnoses, therapeutic targets, and prognosis [5,8]. The DNA methylation in KIRC has been studied for several years [9]. The suppression of *Wnt* antagonists through DNA methylation plays an important role in the proliferation of many RCC cell lines and patient tumor samples [10]. The DNA methylation of several genes, such as *PARVG, PLCB2*, and *RAC2*, are correlated with RCC prognosis [11]. The hypermethylation of cystatin 6 and *LAD1* appears to be associated with poor prognosis and poor response to some antiangiogenic therapies in RCC [12].

With the rapid development of computer technology, an increasing number of biomarkers of various tumors are identified using bioinformatics analysis, and many tumor-associated databases are established [13]. The Cancer Genome Atlas (TCGA) is a public database that includes 33 cancer types and matched clinical data [14]. Studies reveal many genes, such as *CEP55, ACAA1, ACADSB, ALDH6A1*, and *AUH*, that may serve as potential diagnostic and prognostic biomarkers for KIRC [13,15]. The methylation-driven gene is differentially expressed in the control and the disease groups, and the methylation status is negatively correlated with corresponding gene expression value. At present, systemic analysis on methylation-driven genes in KIRC remains limited. In addition, no multivariate prognostic model based on methylation-driven genes especially a clinical prediction model combining DNA methylation data and clinical data exists.

In the present study, we combined the TCGA–KIRC clinical, methylation, and expression data to identify the DNA methylation profiling in KIRC, discover the prognosis-related methylation genes and position, establish methylation prognostic prediction model, and construct an easy trans-omics prognostic nomogram. The findings of the present study can provide valuable data resources for KIRC in clinical and molecular study in the future.

## Methods

### Preparation of dataset

The KIRC methylation data, RNA-seq expression counts, and clinical information were downloaded from the TCGA website (https://cancergenome.nih.gov/). After combining the methylation data, the methylation positions and gene names were matched, and the average methylation level of each gene was calculated (normal samples = 160, tumor samples = 325). The R package DEseq2 (version 1.20.0) was used to standardize the RNA-seq expression counts and obtain the gene expression value matrix. The follow-up time, survival state, age, gender, and tumor stage were extracted from clinical information and used for subsequent analysis. A total of 317 tumor samples were obtained by matching the methylation data, gene expression value, and clinical information matrices in accordance with the sample number. The samples with a survival period of less than 30 days were removed, and a total of 294 tumor samples were obtained for survival analysis.

### Differential methylation analysis

The R package limma (version 3.36.5) was used to calculate the differential methylation between the tumor and the normal groups. The fold change value of methylation levels between the tumor and the normal groups was calculated and then standardized through logarithm. Wilcox test was used for the statistical analysis of methylation data and adjusted. *P* value < 0.05 was used as a cutoff standard. The R package pheatmap (version 1.0.10) was used to visualize differential methylation.

### Calculation of methylation-driven genes

The matched gene expression values matrix, the DNA methylation values of the tumor group, and the DNA methylation values of the normal group were input into the R package MethylMix (version 2.10.2) to obtain the methylation-driven gene. The Wilcox test was used for statistical analysis, and *P* value was adjusted using the Bonferroni method. Adjusted *P* value < 0.05 was used as cutoff standard.

### Gene Ontology (GO) analysis

The GO analysis for all methylation-driven genes was performed using the R package cluster profiler (version 3.8.1) based on org.Hs.eg.dbdatabase (version 3.6.0). The parameters of enrichGO analysis function were: ont = “BP,” pAdjustMethod = “BH,” pvalueCutoff = 0.05. The GO analysis results were visualized using the enrichplot (version 1.2.0) and the GOplot package (version 1.0.2).

### Construction of the methylation prognostic prediction model by using the Lasso regression

The clinical survival information and methylation data of driven genes were integrated in accordance with the sample name. Survival analysis and result visualization were performed using the R package survival (version 2.43-1) and the SurfMiner package (version 0.4.3). The “Handout” method was used for model construction and testing. Briefly, all samples were divided into the training (70%) and the testing (30%) groups by using the hierarchical random method through the “createDataPartition” function in the machine learning R package “caret” (version 6.0-81) to keep each categorical variable of the data in the subset consistent with the original proportion of the overall data, thereby ensuring that the data distribution of the training and the testing groups is consistent. The methylation prediction model in the training group was established as follows. The cv.glmnet function of the machine learning package glmnet (version 2.0-16) was used to calculate the Lasso rank. The glmnet function was used to calculate the Lasso by using the Cox multivariate regression model. The model with the best performance and the least number of independent variables was selected using the best lambda value. Samples were divided into high- and low-risk groups, and the survival rate was further compared using this methylation prognostic prediction model. *P*<0.05 was regarded as the cutoff standard. The predictive efficiency was evaluated using the area under the receiver operating characteristic curve (AUC). This methylation prognostic prediction model was also validated in the testing group.

### Correlation analysis of methylation-driven genes and methylation positions

In accordance with the name of selected methylation-driven genes in the previous methylation prognostic prediction model, the multiple methylation position details of these genes were extracted from the TCGA methylation dataset. The Rcorr function in the Hmisc package (version 4.1-1) was used to calculate the correlation between the expression value of methylation-driven genes and methylation positions. The significant correlations were screened using the absolute value of correlation coefficient > 0.4 and the *P* value of correlation test < 0.05. These correlations were visualized using the Cytoscape software (version 3.7.1).

### Construction of trans-omics prognostic nomogram

The clinical information, such as age, gender, and tumor stage, combined with the score of methylation prognostic model was used to construct the trans-omics prognostic prediction model. The machine learning package caret (version 6.0-81) was used to divide all samples through random stratification. Approximately 70% and 30% of the samples were used as the training and the testing groups, respectively. The rms package (version 5.1-2) was used to build the Cox proportional hazards model, draw the nomogram, and calculate the c-index to evaluate the efficiency of the trans-omics prediction model in the training and the testing groups. In both groups, the machine learning calibration method was used to calculate and draw the calibration curve to evaluate the model’s accuracy.

## Results

### Differential methylation gene analysis

**Figure 1:**
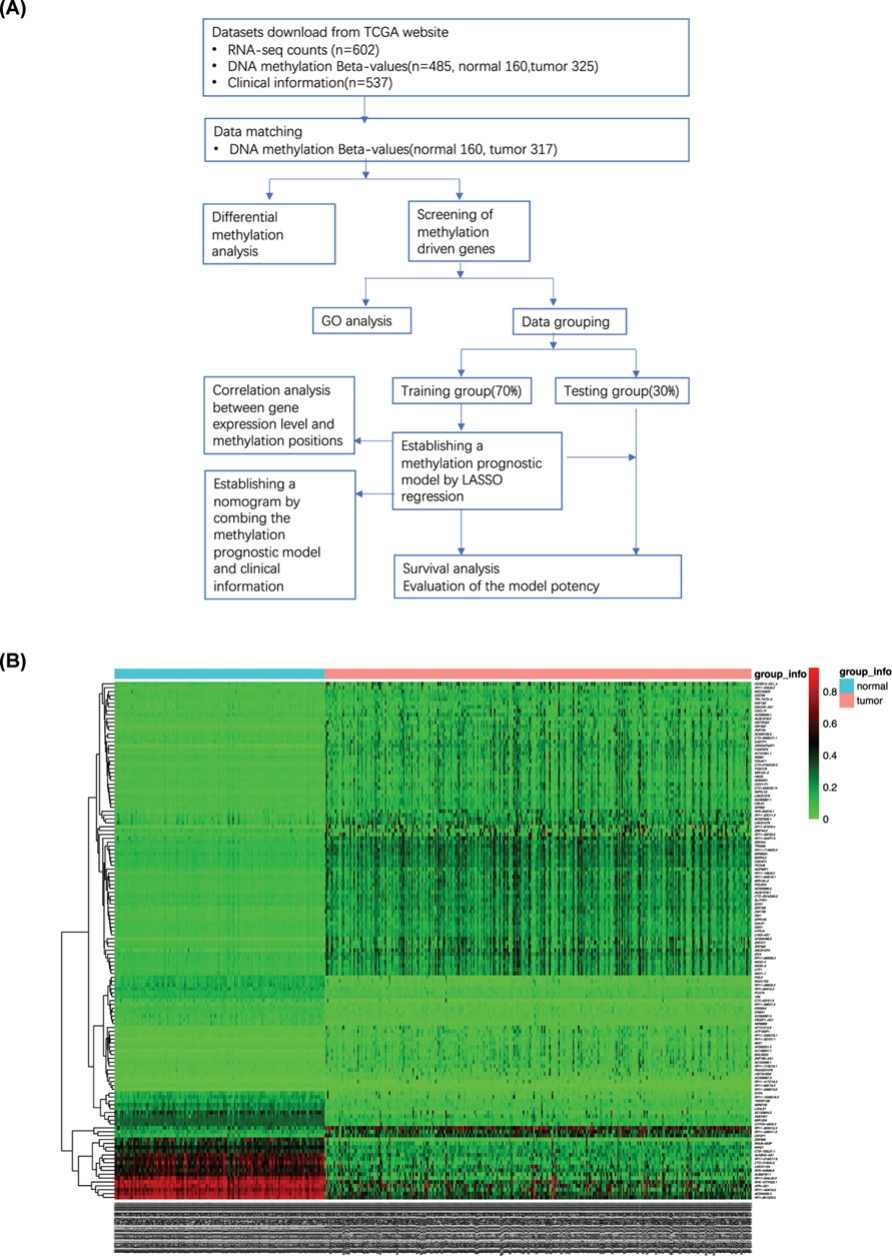
Workflow and hierarchical clustering of differential methylation genes (**A**) Workflow of the experiments. (**B**) Heat map of differential methylation genes between tumor group and normal group. Genes with |log2FC| <1 and adjusted. *P* value < 0.05 is shown in the map. Top band is grouping information (red for tumor group, blue for normal group); left is clustering tree; bottom is sample number; right is the name of the methylation gene.

Figure 1A shows the flow diagram of the present study. We obtained the related RNA-seq counts (*n*=602), DNA methylation β-values (*n*=485, normal = 160, tumor = 325), and clinical information (*n*=537) from TCGA. The methylation of some samples was not tested, and the methylation and the expression data did not have one-to-one correspondence. After matching the methylation data with the expression data in accordance with the sample number, we obtained DNA methylation samples (*n*=477, normal = 160, tumor = 317) to screen methylation-driven genes. Then, we constructed and verified the methylation prognostic model by using machine learning.

A total of 134 differential methylation genes with |log 2 FC| >1 were adjusted. *P* values < 0.05 are shown in the hierarchical clustering heat map (Figure 1B). The methylation levels of the normal and the tumor groups were significantly different, and the hypomethylation and the hypermethylation genes were distinguished evidently by hierarchical clustering. Results also showed that the hypomethylation and the hypermethylation genes contained protein coding genes, such as *CECR6, RNF180, CXCL1P, HIST3H2A*, and *ZNF492*, and noncoding genes, such as *HOXB13–AS1_2, RP11–343J3.2, DGUOK–AS1, AC009506.1*, and *AL021918.2*, which indicated that the extent of methylation was universal for genes.

### Methylation-driven gene discovery and GO analysis

**Figure 2:**
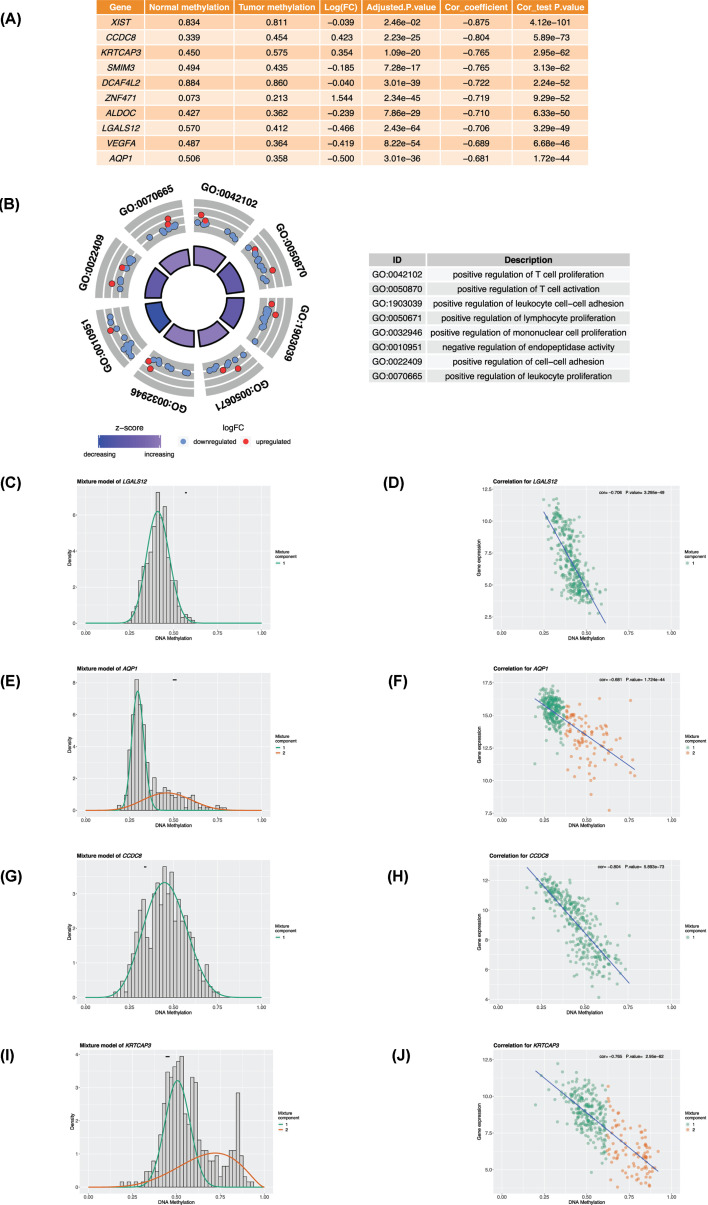
Methylation-driven genes (**A**) Top 10 genes with the highest absolute correlation between the methylation level and expression value. (**B**) GO analysis of all methylation-driven genes. The left figure shows the *Z*-score and gene distribution of the top eight GO terms, and the right figure shows the name of the top eight GO terms. (**C** and **D**) Density map of the representative hypomethylation gene LGALS12 (a single component distribution pattern) and the scatter map of correlation with gene expression value; the black short line in panel (C) represents the average methylation level of the normal group. (**E** and **F**) Density map of the representative hypomethylation gene AQP1 (two-component distribution pattern) and the scatter map of correlation with gene expression value; the black short line in panel (E) represents the average methylation level of the normal group. (**G** and **H**) Density map of the representative hypermethylation gene CCDC8 (a single component distribution pattern) and the scatter map of correlation with gene expression value; the black short line in panel (G) represents the average methylation level of the normal group. (**I** and **J**) Density map of the representative hypermethylation gene KRTCAP3 (two-component distribution pattern) and the scatter map of correlation with gene expression value; the black short line in panel (I) represents the average methylation level of the normal group.

A total of 242 methylation-driven genes were screened using the MethylMix package. The detailed information of all methylation-driven genes is listed in Supplementary Table S1. The top 10 genes with the highest absolute correlation coefficient between methylation state and gene expression value are shown in Figure 2A. These genes were *XIST, CCDC8, KRTCAP3, SMIM3, DCAF4L2, ZNF471, ALDOC, LGALS12, VEGFA*, and *AQP1*. The correlation coefficients were between −0.681 and −0.875 and showed a significant difference (*P*<0.05).

The GO analysis of the 242 methylation-driven genes showed that in biological process, 242 methylation-driven genes was mainly clustered in the activation of immune cells (GO:1903039: positive regulation of T-cell activation), the adhesion of immune cells (GO:0050671: positive regulation of leukocyte cell–cell adhesion), and the proliferation of immune cells (GO:0050870: positive regulation of T-cell proliferation; GO:0010951: positive regulation of mononuclear cell proliferation) (Figure 2B). These biological processes were closely related to tumorigenesis and development.

Figure 2C–J shows the typical gene methylation pattern of the top 10 genes, and Figure 2A,C shows the methylation density distribution of the *LGALS12* gene, which was hypomethylated in the tumor group with simple methylation component. Figure 2D shows the correlation coefficient (*r* = −0.706, *P*=3.295e–49) between the expression value of *LGALS12* and its methylation level. *AQP1* also had low methylation in the tumor group but had two components with different distributions. The correlation coefficient between the expression value and the methylation state was −0.681 (*P* value = 1.724e–44; Figure 2E,F). Figure 2G shows that *CCDC8* was a hypermethylation gene with a single methylation component in the tumor group. Figure 2H shows the correlation coefficient (*r* = −0.804, *P*=5.893e–73) between the expression value of *CCDC8* and the methylation level. The *KRTCAP3* gene was also hypermethylated in the tumor group with two components in different distribution. The correlation coefficient between the expression value and the methylation level was −0.765 (*P*=2.95e–62, Figure 2I,J).

### Construction and evaluation of the methylation prognostic prediction model by using machine learning

**Figure 3:**
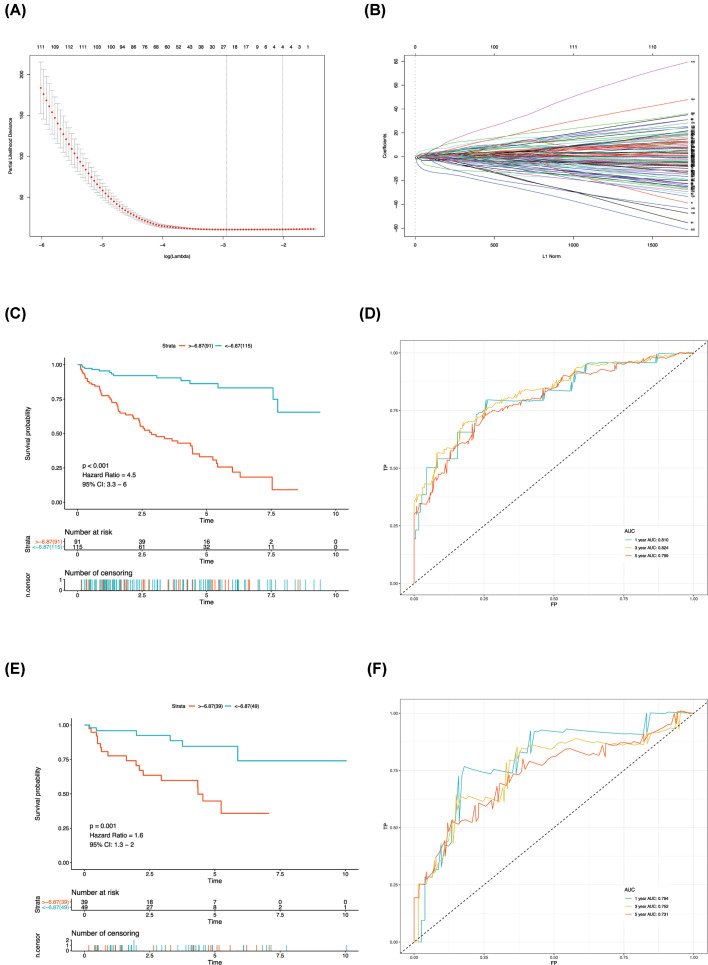
Establishment and validation of methylation prognostic prediction model via machine learning (**A**) Selection of the tuning parameter (*λ*) in the Lasso model. The *Y*-axis represents partial likelihood deviance, and the lower *X*-axis represents the log (*λ*). Numbers along the upper *X*-axis represent the average number of predictors. Red dots indicate average deviance values for each model with a given *λ*, where the model provides its best fit to data. (**B**) Lasso coefficient profiles of the survival-associated methylations. The gray dotted vertical line is the value selected using 10-fold cross-validation in A. (**C**) This part shows the Kaplan–Meier (K-M) curves for high- and low-risk groups in the training set. (**D**) ROC curves for patients under training set at 1, 3, and 5 years. (**E**) This part shows the K-M curves for high- and low-risk groups in the testing set. (**F**) ROC curves for patients under testing set at 1, 3, and 5 years. AUC, area under the ROC curve.

Samples with survival period less than 30 days were removed, and the remaining samples were stratified to obtain the training (*n*=206) and the testing (*n*=88) groups. First, the Lasso method was used to screen the variables and establish the survival prognosis model in the training group. As shown in Figure 3A,B, the Lasso logistic regression was performed in 242 methylation-driven genes. Certain coefficients were accurately reduced to 0 by forcing the total absolute value of the regression coefficients to be less than the constant value, and the most powerful prognostic predictors were selected. The survival prognosis model included four gene methylation data, namely, *FOXI2, USP44, EVI2A*, and *TRIP13* with Lasso coefficients of 1.7373362, 0.4491624, −1.7746901, and −3.2954915, respectively.

We used the present model to score and divide the training group into high- and low-risk groups in accordance with the best segmentation point. Compared with the survival rate of the two groups, the Kaplan–Meier (K-M) curve showed that the survival rate of the high-risk group was significantly lower than that of the low-risk group (*P*<0.001, Figure 3C). The ROC curve was used to evaluate the prediction efficiency. The areas of 1-, 3-, and 5-year survival rates were 0.810, 0.824, and 0.799, respectively. Results indicated that this model had a good prediction ability for the training group (Figure 3D).

Similarly, we used the same model to score the testing group and compared the survival rate of the high- and the low-risk groups. The K-M curve also showed that the survival rate of the high-risk group was significantly lower than that of the low-risk group (*P*=0.001, Figure 3E). The ROC curve indicated that the 1-, 3-, and 5-year survival rates were 0.794, 0.752, and 0.731, respectively. The prediction ability of the model was also desirable for the testing group.

### Analysis of methylation positions of the key genes in the prediction model

**Figure 4:**
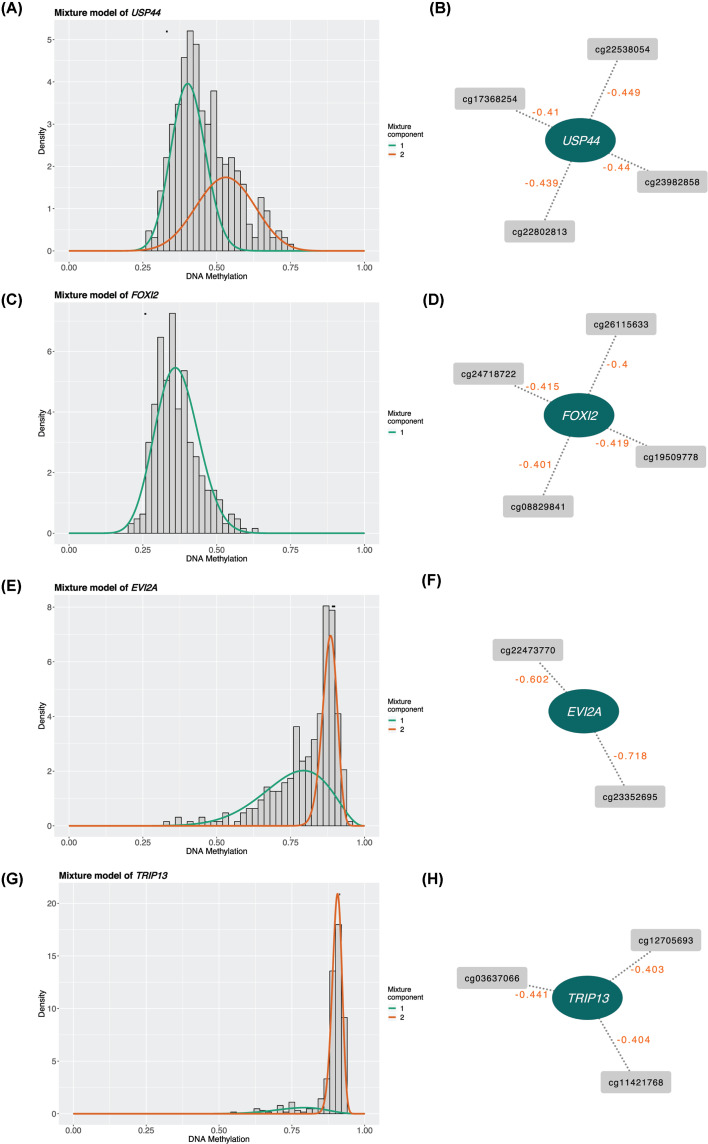
Correlation analysis of genes and methylation position in the prognosis model (**A**) Methylation density map of USP44 gene. (**B**) Correlation analysis of USP44 gene expression level and methylation position (the value on the dotted line is the correlation coefficient, correlation test *P*<0.05). (**C**) Methylation density map of FOXI2 gene. (**D**) Correlation analysis of foxi2 gene expression level and methylation site position (the value on the dotted line is the correlation coefficient, correlation test *P*<0.05). (**E**) EVI2A gene methylation density map. (**F**) Correlation analysis of EVI2A gene expression level and methylation position (the value on the dotted line is the correlation coefficient, correlation test *P*<0.05). (**G**) Methylation density map of TRIP13 gene. (**H**) Correlation analysis of TRIP13 gene expression level and methylation position (the value on the dotted line is the correlation coefficient, correlation test *P*<0.05).

We further investigated the methylation and the main methylation positions of the four key genes in the prediction model. Results showed that *USP44* was a hypermethylation gene in the tumor group (Figure 4A), and the most related methylation positions were cg22538054, cg23982858, cg22802813, and cg17368254 (Figure 4B). *FOXI2* was also a hypermethylation gene (Figure 4C), and the most related methylation positions were cg19509778, cg24718722, cg08829841, and cg26115633 (Figure 4D). For the low methylation gene (Figure 4E,G), the most relevant methylation positions of *EVI2A* were cg2332595 and cg22473770 (Figure 4F), and the most relevant methylation positions of *TRIP13* were cg03637066, cg11421768, and cg12705693 (Figure 4H). These selected methylation positions will contribute to further identify the role of these genes in KIRC development.

### Easy trans-omics prognostic nomogram combined with methylation prediction model and clinical information

**Figure 5:**
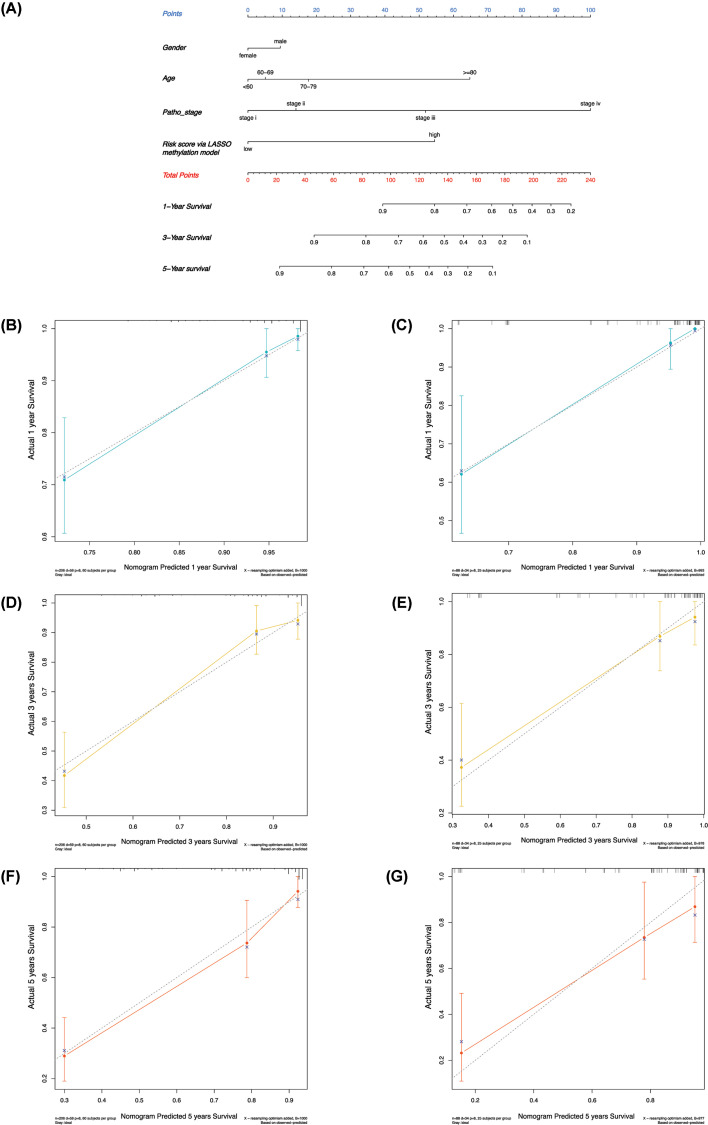
Combining the score of methylation-driven gene prognostic model and clinical information to construct nomogram for prognosis prediction (**A**) Nomogram of overall survival (OS) in KIRC. The top points are the scale used for scoring; clinical indexes and methylation-driven gene prognosis model scores can correspond to the top scale scores. Add the scores of each predictor to obtain the total score. The total score on the total point scale is located, and then the coordinates corresponding on the probability scale at the bottom of the figure are used to obtain 1-, 3-, and 5-year probability of survival. (**B–G**) Calibration curves of the nomogram in the training and testing group of KIRC. (B) Prediction of 1-year OS in the training group of KIRC. (C) Prediction of 1-year OS in the testing group of KIRC. (D) Prediction of 3-year OS in the training group of KIRC. (E) Prediction of 3-year OS in the testing group of KIRC. (F) Prediction of 5-year OS in the training group of KIRC. (G) Prediction of 5-year OS in the testing group of KIRC.

Tumor prognosis was closely related to clinical stage and other indicators. We combined several key clinical indicators, such as age, gender, tumor stage, and methylation model score, to create a simple prognostic nomogram and establish a comprehensive and easy prediction system. Figure 5A shows that the training group had the largest weight of the tumor stage, same extent of age and methylation prognosis score, and small gender effect. Through the score of these indicators, the 1-, 3-, and 5-year survival rates were easily obtained by calculating the total scores and the corresponding survival rate. The C-indices of the training and the testing groups were 0.8015 and 0.8389, respectively, which indicated that the prediction system was effective and would be verified in other cases. The nomogram displayed high levels of accuracy to predict the 1-, 3-, and 5-year overall survival of patients with KIRC in the training and the testing groups, as shown in the calibration curves (Figure 5B–G). Therefore, the prognostic nomogram works accurately for patients with KIRC.

## Discussion

In our study, we have found many differential methylation genes and positions. Methylation occurs in coding and noncoding genes. For coding genes, DNA methylation has been observed in the suppression of specific tumor suppressor genes [16]. The methylation loss of *CDKN2A* and *CDKN2B* is demonstrated in myeloma patients [16]. The hypomethylation of *SALL4*, a member of the zinc-finger transcription factor gene family, is responsible for the aberrant expression of *SALL4* in B-ALL [17]. Another level of methylation is the influence of noncoding genes on protein expression regulation. microRNAs and ncRNAs may have important roles [13]. The methylation-mediated loss of miR-34b/c can up-regulate its oncogenic target genes [18,19]. The hypermethylation of mir-127 and mir-125b-1 in breast cancer is closely associated with tumor metastasis [20]. The methylation of noncoding genes has an important prospect, and their direct or indirect regulation with gene expression needs to be studied. Our study also indicates that the methylation of coding and noncoding genes extensively exists in KIRC.

We have used the negative correlation between the gene methylation level and expression value to select methylation-driven genes via the R package MethylMix, a generally accepted method [21,22]. Otherwise, establishing the corresponding relationship between methylation and expression value is difficult. In accordance with this criterion, more than 200 methylation-driven genes are identified. Their functions are concentrated on immunoregulation, such as the activation, proliferation, and adhesion of immune cells, which are closely related to the mechanism of tumorigenesis and development. KIRC has a high level of tumor-infiltrating immune cells [23]. The activated CD8+T cells are associated with the prognosis of many cancers, including RCC, and the infiltrating CD4+ T cells can regulate RCC cell proliferation by modulating [24]. Our GO results suggest that these methylation-driven genes can be significant in immune infiltration in KIRC.

In addition, we have found that the methylation of some genes has different distribution patterns in different samples, indicating that different subtypes may be present in KIRC. Previous studies have reported that several genes, such as *NTHL1, ZCCHC24*, and *SNX1b*, have different methylation status in different subtypes of breast cancer [25]. These features need further study, and these genes may become potential clinical biomarkers for KIRC subtyping. We have calculated the average methylation level of the genes in the present study. However, the methylation levels of different methylation positions in the same gene are different. Our analysis also provides several methylation positions with the largest correlation among key genes in the model. Further investigation on these positions can provide evidence for single-gene methylation.

The Lasso regression, a kind of machine learning method, is suitable for the multivariate selection. This method shrinks the regression coefficients, thereby effectively selecting important predictors and the established model that can compress the number of variables as much as possible [26,27]. In our study, we have used the selected methylation-driven genes as variables and established a multivariate prognostic model by using the machine learning method. The model only contains four methylation genes and is relatively simple. These genes and their methylation have not been reported in KIRC. For the functions of these genes, *USP44* is related to proliferation, migration and invasion, induced apoptosis, and arrested cell cycle in the G2/M phase in the established glioma cell lines [28]. *FOXI2* methylation may be associated with increased risk of oral and colorectal cancers [29,30]. *EVI2A* hypomethylation may be associated with head and neck squamous cell carcinomas [31]. *TRIP13* plays an indispensable role in cell progression, contributing to tumorigenesis and drug resistance [32]. The functions of these genes are closely related to tumors, further indicating that they may play important roles in KIRC. By using these gene methylation statuses as score criterion, we can easily distinguish high- and low-risk patients through this model. The model’s predictive efficiency (i.e. AUC) is also satisfactory, which is verified in the testing group. The change in the DNA methylation pattern may be a good indicator of tumorigenesis and development. Several studies have mentioned the clinical application of multivariate predictive model through DNA methylation. Studies have reported the methylation profiles of HCC tumor DNA and successfully constructed a diagnostic prediction model to predict prognosis and survival rate [33]. Another study has also constructed a survival prognosis model by using the DNA methylation signature in ovarian serous cystadenocarcinoma, which shows high sensitivity and specificity to predict the prognostic survival of patients [34]. Our results demonstrate that the model shows a superior performance for prediction and has potential clinical application.

Tumor prognosis cannot be determined using a single factor. We combined the methylation prognostic prediction model with the clinical information of patients and constructed an easy and effective trans-omics prognostic nomogram to further study the clinical prediction model. Trans-omics integrates clinical and molecular multiomics [35]. Many research studies combine biomarkers and clinical indicators to construct the clinical model. In lung cancer, serum biomarkers ProGRP, CEA, SCC, and CYFRA21-1 are combined with clinical information to construct patient and nodule risk models [36]. A prognostic prediction model reveals that C1QTNF3 is a promising biomarker for prostate cancer [37]. Our trans-omics prognostic nomogram is accurate, simple, and easy to apply. We have constructed this novel nomogram in KIRC, which has great value for the prediction of survival rate.

Recently, studies report a few prognostic models related to methylation in KIRC. The model reported by Xu et al. is based on five methylated CpG sites without trans-omics data [38]. In Guang Chen’s study, the top 15% of the nodes in the enriched pathway networks are selected to screen the methylated genes related to prognosis. However, in the process of variable screening, some important information may be missed. Moreover, the model’s performance has not been evaluated [39]. Evelönn et al. has built a model from local patients’ CpG methylation data combined with CNV data and only used TCGA data for validation. This model has many complicated parameters. which may lead to limitation for clinical application [1]. The above models are completely different from the model established in our study. The model built by Hu et al. is similar to ours [40]. However, methylation-driven genes are not used for variable screening. Seven genes are incorporated in their model, whereas only four genes are incorporated in our model. Moreover, nonsilent mutations in VHL are incorporated in their model, which may lead to increased difficulty of practice. The factor of gender is not incorporated in their model. The AUC values of their model in the testing dataset are only 0.677, 0.66, and 0.71 (1-, 3-, and 5-year survival rates, respectively), whereas the AUC values of our model are 0.794, 0.752, and 0.731 (1-, 3-, and 5-year survival rates, respectively). Therefore, the model discovered in the present study is simple and efficient.

## Conclusion

Several limitations were reported in the present study. First, the external data were not available for further verification. Second, the analysis results should be supported and verified by clinical and experimental tests. In general, the present study was the first to reveal the overall perspective of methylation-driven genes in KIRC. We used the machine learning method to establish a multivariate methylation prognostic prediction model and combined with clinical information to build the trans-omics prognostic nomogram. The model discovered in the present study is novel, simple, and efficient. These results can help in the accurate evaluation of the prognosis of KIRC patients and provide new clues and data resources for the further study of the pathogenesis and the development of the disease.

## Supplementary Material

Supplementary Table S1

## Data Availability

All data generated or analyzed during this study are available from the corresponding author on reasonable request.

## Abbreviations

CpG: Cytosine–phosphate–guanine
KIRC: Kidney renal clear cell carcinoma
K-M: Kaplan–Meier
OS: Overall survival
TCGA: The Cancer Genome Atlas
