# Supplementary material for: Novel prognostic prediction model constructed through machine learning on the basis of methylation-driven genes in kidney renal clear cell carcinoma
Source: Biosci Rep. 2020 Jul 21;40(7):BSR20201604. doi: 10.1042/BSR20201604 (PMC7374278; doi:10.1042/BSR20201604)
Supplement: Supplementary Table S1 [file BSR-2020-1604_supp.pdf]

gene	normalMean	TumorMean	logFC	pValue	adjustP	cor	corPavlue
LUCAT1	0.603413731	0.46292034	-0.3823836	2.79E-69	7.41E-67	-0.4666416	1.51E-18
RARRES3	0.46420818	0.32530655	-0.5129721	1.48E-67	3.94E-65	-0.4098343	2.86E-14
MOB3A	0.706080858	0.55350506	-0.3512369	5.97E-67	1.59E-64	-0.4571732	8.90E-18
LGALS12	0.569889202	0.41249061	-0.4663202	9.15E-67	2.43E-64	-0.706444	3.29E-49
NNMT	0.501292611	0.38607098	-0.3767869	1.54E-66	4.11E-64	-0.4262711	1.99E-15
SIRPA	0.798533712	0.64364475	-0.3110887	6.79E-66	1.80E-63	-0.4114688	2.21E-14
CD68	0.46134295	0.27557967	-0.7433702	1.42E-65	3.77E-63	-0.4072749	4.27E-14
SLC16A3	0.513635602	0.37012782	-0.4727216	2.69E-65	7.15E-63	-0.5230814	1.16E-23
NAA11	0.743725514	0.69338446	-0.1011148	3.55E-65	9.43E-63	-0.503988	7.98E-22
LGALS2	0.494354182	0.32703321	-0.5961079	1.33E-64	3.53E-62	-0.3188293	6.38E-09
SLAMF8	0.46725757	0.34621402	-0.4325539	1.44E-64	3.84E-62	-0.5010551	1.49E-21
TNFAIP6	0.879446038	0.64200047	-0.4540207	1.48E-64	3.94E-62	-0.6001805	2.10E-32
TNFSF10	0.478160102	0.33347129	-0.5199312	2.76E-64	7.33E-62	-0.4593016	6.01E-18
TIGIT	0.830030159	0.72947171	-0.1863117	4.39E-64	1.17E-61	-0.5219856	1.49E-23
ITIH4	0.747405007	0.64254747	-0.2180872	7.51E-64	2.00E-61	-0.3517301	1.16E-10
SMYD4	0.766321089	0.59747509	-0.3590704	1.39E-63	3.71E-61	-0.3818727	1.91E-12
ZNF728	0.069052938	0.22959093	1.73329096	2.35E-63	6.25E-61	-0.3578481	5.21E-11
REG1A	0.739836789	0.58409803	-0.3409965	1.03E-61	2.75E-59	-0.3196052	5.84E-09
C15orf53	0.745464744	0.629933	-0.2429417	1.87E-61	4.99E-59	-0.4136535	1.56E-14
SHMT2	0.523300648	0.36508092	-0.5194238	1.50E-60	4.00E-58	-0.6249737	9.49E-36
TM4SF1	0.639648387	0.5328248	-0.2636178	4.12E-60	1.09E-57	-0.4161968	1.04E-14
FOXI2	0.258266384	0.36578576	0.50213903	5.24E-60	1.40E-57	-0.3485504	1.74E-10
C11orf21	0.682091498	0.53137731	-0.3602287	7.58E-60	2.02E-57	-0.3315303	1.44E-09
NFE2L3	0.365742577	0.2610045	-0.4867539	1.00E-59	2.66E-57	-0.606474	3.17E-33
SPINK13	0.688577409	0.35894309	-0.9398637	3.30E-59	8.78E-57	-0.3230408	3.92E-09
NKAPL	0.346703369	0.47422251	0.45186228	1.75E-58	4.65E-56	-0.4954955	4.82E-21
C19orf33	0.431767763	0.29695619	-0.5400054	2.08E-58	5.52E-56	-0.5577664	2.58E-27
FMNL1	0.554424772	0.45367272	-0.2893398	2.12E-58	5.65E-56	-0.4278373	1.54E-15
IFI16	0.375873102	0.26036027	-0.5297363	3.20E-58	8.50E-56	-0.561286	1.04E-27
LINC00944	0.339187101	0.1907369	-0.8304973	9.28E-58	2.47E-55	-0.5962461	6.70E-32
CARD16	0.658577491	0.5445459	-0.2742995	9.93E-58	2.64E-55	-0.4643568	2.33E-18
HAVCR1	0.500128788	0.3995661	-0.3238655	1.69E-57	4.49E-55	-0.3534627	9.25E-11
RNASE2	0.650573905	0.5518782	-0.2373631	2.77E-57	7.38E-55	-0.4564384	1.02E-17
TRAF1	0.571773172	0.44849348	-0.3503559	3.07E-57	8.17E-55	-0.4112864	2.27E-14
LRRC25	0.404837168	0.26285515	-0.6230738	6.31E-57	1.68E-54	-0.4787784	1.44E-19
VAMP5	0.343430316	0.23419422	-0.5523119	6.98E-57	1.86E-54	-0.4259749	2.10E-15
ARHGAP30	0.615781928	0.49354342	-0.3192425	1.07E-56	2.84E-54	-0.5043288	7.42E-22
LINC01315	0.303070483	0.19934676	-0.6043732	1.93E-56	5.14E-54	-0.4697598	8.35E-19
VEGFA	0.486542973	0.3638322	-0.419294	3.09E-56	8.22E-54	-0.6887196	6.68E-46
TGIF2LX	0.764022827	0.66108915	-0.2087709	3.50E-56	9.30E-54	-0.3384313	6.20E-10
PROM2	0.714331837	0.80932774	0.18012962	3.70E-56	9.83E-54	-0.5493406	2.18E-26
SLC2A3	0.328468121	0.20303294	-0.6940396	6.52E-56	1.74E-53	-0.3498868	1.47E-10
RAB25	0.273984134	0.39844271	0.54027996	7.62E-56	2.03E-53	-0.6441051	1.53E-38
IL2RA	0.746419504	0.64068786	-0.220365	7.71E-56	2.05E-53	-0.3983514	1.68E-13
CYP3A5	0.445444972	0.3177299	-0.4874463	9.42E-56	2.50E-53	-0.4548098	1.37E-17
LGALSL	0.891874438	0.77814579	-0.1968001	1.85E-55	4.93E-53	-0.4055536	5.58E-14
HOXB-AS3	0.305672691	0.41771855	0.45054355	3.19E-55	8.48E-53	-0.5164599	5.18E-23
BST2	0.548760294	0.37093929	-0.564993	4.74E-55	1.26E-52	-0.6754838	1.39E-43
SLPI	0.549901683	0.33852839	-0.6998969	5.24E-55	1.39E-52	-0.5832288	2.79E-30
TRAF3IP3	0.782878184	0.63032998	-0.3126806	7.37E-55	1.96E-52	-0.4638164	2.58E-18
CD28	0.73503526	0.62482725	-0.2343561	7.54E-55	2.01E-52	-0.4201346	5.48E-15
HHLA2	0.41757745	0.3042355	-0.4568553	1.32E-54	3.51E-52	-0.4811527	8.99E-20
CASS4	0.569734852	0.37306724	-0.610855	1.72E-54	4.58E-52	-0.4560124	1.10E-17
ZNF454	0.121253798	0.24591484	1.02012884	2.07E-54	5.51E-52	-0.3712624	8.51E-12
LINC00601	0.91118977	0.76963667	-0.243574	6.69E-54	1.78E-51	-0.4616539	3.88E-18
ACSM5	0.585471485	0.44877132	-0.3836184	9.38E-54	2.50E-51	-0.4738956	3.75E-19
C5orf46	0.74853784	0.68799005	-0.1216875	1.40E-53	3.73E-51	-0.3248056	3.19E-09
ZNF492	0.034159611	0.14745183	2.10988022	1.73E-53	4.59E-51	-0.5006175	1.64E-21
AC005498.3	0.038428209	0.21165219	2.46145776	3.17E-53	8.44E-51	-0.4383499	2.57E-16
PRF1	0.738406973	0.67269262	-0.1344687	5.51E-53	1.47E-50	-0.3761601	4.30E-12

SLC17A3	0.787911561	0.62211539	-0.3408515	7.79E-53	2.07E-50	-0.4010563	1.12E-13
USP44	0.331072181	0.45726179	0.46587459	1.11E-52	2.96E-50	-0.3273091	2.38E-09
TCF19	0.821530167	0.71618342	-0.1979844	1.75E-52	4.66E-50	-0.4219578	4.07E-15
ZNF582	0.087459064	0.19594396	1.16376132	2.01E-52	5.36E-50	-0.5588672	1.95E-27
LBP	0.636082575	0.55879045	-0.1869067	2.04E-52	5.41E-50	-0.4734149	4.12E-19
HOXB6	0.641467117	0.8655044	0.43216584	3.34E-52	8.88E-50	-0.3729258	6.76E-12
TRAV36DV7	0.892029022	0.72440919	-0.3002858	3.34E-52	8.88E-50	-0.4345223	4.96E-16
ZNF503-AS:	0.350581409	0.45350907	0.3713819	6.15E-52	1.64E-49	-0.423806	3.00E-15
ADAM28	0.51074373	0.36305557	-0.4924092	1.29E-51	3.42E-49	-0.3368071	7.57E-10
CCDC181	0.237169269	0.32030302	0.43352033	1.48E-51	3.93E-49	-0.3309259	1.54E-09
BATF	0.746125289	0.67361377	-0.1474963	5.42E-51	1.44E-48	-0.5300522	2.31E-24
ZNF300P1	0.227291693	0.34120468	0.58609247	2.82E-50	7.50E-48	-0.5982916	3.67E-32
AGXT2	0.593529127	0.47810178	-0.3120011	2.85E-50	7.58E-48	-0.4293692	1.19E-15
HOXA-AS2	0.333507179	0.46090173	0.46674135	5.03E-50	1.34E-47	-0.3648513	2.04E-11
GGT6	0.442601302	0.59049458	0.41591613	5.83E-50	1.55E-47	-0.4205605	5.11E-15
SLAMF7	0.480900478	0.38733881	-0.3121423	7.58E-50	2.02E-47	-0.3298665	1.75E-09
ABHD11-AS	0.450732899	0.33534072	-0.4266451	8.33E-50	2.21E-47	-0.5566315	3.46E-27
PRR15L	0.406948663	0.51547659	0.34106009	1.16E-49	3.10E-47	-0.5578931	2.50E-27
CCL20	0.878378137	0.78429305	-0.1634493	1.29E-49	3.44E-47	-0.4728324	4.62E-19
MTHFR	0.300094332	0.18282898	-0.7149213	1.83E-49	4.86E-47	-0.4643856	2.32E-18
SASH3	0.628031367	0.52472028	-0.2592881	1.86E-49	4.96E-47	-0.6266797	5.45E-36
TINAG	0.568616726	0.43522642	-0.3856904	3.56E-49	9.47E-47	-0.349579	1.53E-10
ITGB8	0.743209	0.59541967	-0.3198611	4.08E-49	1.08E-46	-0.3433946	3.35E-10
SP140L	0.405063867	0.30206347	-0.4232977	4.71E-49	1.25E-46	-0.5906741	3.38E-31
ITGA2	0.453030365	0.34745543	-0.3827798	5.40E-49	1.44E-46	-0.3227275	4.07E-09
HSPB7	0.738021882	0.84198176	0.19012539	7.53E-49	2.00E-46	-0.3723202	7.35E-12
VCAM1	0.566018259	0.4532341	-0.3205922	1.49E-48	3.97E-46	-0.4051772	5.91E-14
RBBP8NL	0.639970249	0.7268934	0.18373897	7.61E-48	2.02E-45	-0.4724936	4.93E-19
ZNF471	0.073231016	0.21349966	1.54370709	8.78E-48	2.34E-45	-0.7192633	9.29E-52
OSM	0.698762444	0.56101368	-0.3167661	1.14E-47	3.02E-45	-0.3965404	2.21E-13
ANXA2R	0.492990142	0.2839234	-0.7960571	1.26E-47	3.35E-45	-0.3304583	1.63E-09
ZNF577	0.203912031	0.33222661	0.70422075	1.41E-47	3.74E-45	-0.6527374	7.19E-40
SLC12A3	0.736057198	0.8036557	0.12675969	2.35E-47	6.24E-45	-0.4559657	1.11E-17
LINC01233	0.19542676	0.32651507	0.74052155	3.95E-47	1.05E-44	-0.3678252	1.36E-11
ZNF208	0.211850147	0.31471135	0.57098508	4.42E-47	1.18E-44	-0.5774192	1.40E-29
AC009506.1	0.081187836	0.16960409	1.06283546	1.79E-46	4.77E-44	-0.4100549	2.76E-14
RDH5	0.533536739	0.34974811	-0.6092714	2.03E-46	5.39E-44	-0.3434247	3.33E-10
ZNF300	0.240389324	0.35112453	0.54660996	3.22E-46	8.57E-44	-0.536987	4.47E-25
SLC15A2	0.381293772	0.49886492	0.38774624	3.68E-46	9.78E-44	-0.3189139	6.32E-09
CLEC2B	0.515210136	0.37612085	-0.4539647	3.71E-46	9.88E-44	-0.416978	9.14E-15
LINC01093	0.655893967	0.59980569	-0.1289674	1.74E-45	4.64E-43	-0.3334503	1.14E-09
NR0B2	0.592953439	0.67839869	0.19421456	6.07E-45	1.61E-42	-0.3393151	5.56E-10
MIR210HG	0.629817026	0.46769215	-0.4293735	1.81E-44	4.81E-42	-0.567899	1.83E-28
CMTM3	0.299175332	0.17403063	-0.78165	4.48E-44	1.19E-41	-0.4392855	2.18E-16
RNF180	0.056218212	0.11715946	1.05936404	5.90E-44	1.57E-41	-0.4064719	4.83E-14
C8orf22	0.593553991	0.48390064	-0.2946684	2.57E-43	6.82E-41	-0.4937908	6.88E-21
PGA5	0.738977334	0.59850951	-0.3041559	3.86E-43	1.03E-40	-0.463469	2.76E-18
TM4SF19	0.66596184	0.56475852	-0.2378054	7.41E-43	1.97E-40	-0.4751851	2.92E-19
SFN	0.763673497	0.67699929	-0.1738016	1.61E-42	4.28E-40	-0.6753925	1.44E-43
AC008074.1	0.418272069	0.30794957	-0.4417476	1.99E-42	5.29E-40	-0.3328349	1.23E-09
SOCS3	0.599482555	0.42216016	-0.5059273	2.07E-42	5.50E-40	-0.3330101	1.20E-09
RGS1	0.860225296	0.75689996	-0.1846119	6.13E-42	1.63E-39	-0.3415616	4.21E-10
S1PR4	0.733465481	0.61699389	-0.2494729	9.00E-42	2.39E-39	-0.4577405	8.02E-18
SLC9A3R1	0.569187274	0.43703718	-0.3811474	9.18E-42	2.44E-39	-0.5736074	3.96E-29
DCAF4L2	0.884337858	0.85994784	-0.0403485	1.13E-41	3.01E-39	-0.7222681	2.24E-52
PES1P1	0.822454056	0.79665977	-0.0459714	1.42E-41	3.79E-39	-0.4526114	2.05E-17
AVPR1B	0.235883274	0.18551996	-0.3464987	3.73E-41	9.92E-39	-0.5132841	1.05E-22
ZSCAN1	0.246307614	0.32003971	0.37778969	4.87E-41	1.29E-38	-0.4677388	1.23E-18
GPR65	0.857761123	0.75648884	-0.1812571	5.77E-41	1.54E-38	-0.4562845	1.05E-17
HOXD3	0.326553916	0.42138659	0.36782319	6.72E-41	1.79E-38	-0.403552	7.60E-14
CMYA5	0.286352092	0.39485771	0.46354272	3.70E-40	9.85E-38	-0.3488151	1.68E-10

SLC19A2	0.690304141	0.79941428	0.2117112	6.20E-40	1.65E-37	-0.4479693	4.73E-17
AC003984.1	0.585844	0.47261258	-0.3098585	3.38E-39	8.98E-37	-0.5401115	2.11E-25
ANGPTL1	0.581738251	0.72721955	0.32202082	5.77E-39	1.54E-36	-0.3934049	3.54E-13
AQP1	0.505841897	0.35768555	-0.4999947	1.13E-38	3.01E-36	-0.6807421	1.72E-44
TACSTD2	0.164821032	0.29798397	0.85433439	1.16E-38	3.10E-36	-0.5924157	2.04E-31
BHMT	0.450426706	0.34480969	-0.385492	1.55E-38	4.12E-36	-0.644024	1.57E-38
LST1	0.521547953	0.45588666	-0.1941247	4.40E-38	1.17E-35	-0.3549585	7.61E-11
UGT1A8	0.865940541	0.80827925	-0.0994142	9.62E-38	2.56E-35	-0.4749987	3.03E-19
ZNF418	0.184993317	0.26101813	0.49667684	1.90E-37	5.05E-35	-0.5995832	2.51E-32
PRSS44	0.135402133	0.20974456	0.63138291	2.24E-37	5.95E-35	-0.3178941	7.10E-09
PABPC1P4	0.323802199	0.46417636	0.51956026	2.43E-37	6.45E-35	-0.3258258	2.83E-09
SLC39A5	0.614480658	0.50529684	-0.2822364	5.97E-37	1.59E-34	-0.6387135	9.80E-38
TXLNB	0.283202792	0.17913812	-0.6607631	1.06E-36	2.82E-34	-0.3397131	5.29E-10
LTA	0.746499583	0.68906919	-0.1154926	1.50E-36	4.00E-34	-0.5858948	1.32E-30
DAPP1	0.648364709	0.57347092	-0.1770852	4.83E-36	1.28E-33	-0.3392222	5.62E-10
UCN2	0.826689768	0.73501956	-0.1695634	2.67E-34	7.10E-32	-0.317921	7.08E-09
CD3D	0.792525623	0.71325584	-0.1520379	2.74E-34	7.29E-32	-0.3975591	1.90E-13
RXFP4	0.433628602	0.55572022	0.35789881	3.37E-34	8.97E-32	-0.3500917	1.43E-10
SENP8	0.907836002	0.81642779	-0.1531064	4.18E-34	1.11E-31	-0.3829411	1.64E-12
ABCA4	0.5627749	0.60531026	0.10511683	4.37E-34	1.16E-31	-0.6577387	1.17E-40
IL2RG	0.625111885	0.5030587	-0.3133877	6.60E-34	1.76E-31	-0.4235171	3.15E-15
OTOR	0.828749203	0.7942184	-0.0613998	1.68E-33	4.47E-31	-0.3994781	1.42E-13
ZBTB42	0.49830346	0.42624395	-0.2253452	1.86E-33	4.95E-31	-0.6279689	3.57E-36
CSAG1	0.770108973	0.72224029	-0.0925837	2.23E-33	5.93E-31	-0.4464455	6.20E-17
DOPEY2	0.594172502	0.55608875	-0.0955667	9.11E-33	2.42E-30	-0.5773893	1.41E-29
TMEM173	0.32136467	0.23117771	-0.475209	1.51E-32	4.02E-30	-0.6140384	3.08E-34
TNFSF4	0.671183832	0.59762995	-0.1674555	2.74E-32	7.30E-30	-0.3343908	1.02E-09
C19orf67	0.563794782	0.46319288	-0.283557	6.02E-32	1.60E-29	-0.5738085	3.75E-29
CKMT2	0.510507092	0.61570524	0.27030885	6.65E-32	1.77E-29	-0.5764546	1.82E-29
KRT15	0.678397751	0.61912446	-0.1319019	7.86E-32	2.09E-29	-0.4802607	1.07E-19
PFN1	0.79188816	0.61048626	-0.3753379	1.43E-31	3.80E-29	-0.3280023	2.19E-09
LY96	0.634642177	0.55372438	-0.1967754	1.59E-31	4.23E-29	-0.6545933	3.68E-40
AOC1	0.573344978	0.52749797	-0.1202379	1.64E-31	4.37E-29	-0.3190586	6.22E-09
LAIR1	0.43272562	0.35988374	-0.2659216	2.72E-31	7.24E-29	-0.3558909	6.74E-11
ALDOC	0.426561686	0.36155662	-0.2385328	2.96E-31	7.86E-29	-0.7101166	6.33E-50
DQX1	0.773972926	0.71661213	-0.1110906	6.56E-31	1.74E-28	-0.4625169	3.30E-18
TOX3	0.313560129	0.3524617	0.16872439	1.09E-30	2.90E-28	-0.3398134	5.23E-10
HP	0.538989241	0.4794643	-0.1688331	1.27E-30	3.38E-28	-0.544492	7.25E-26
C11orf53	0.726825242	0.69141705	-0.0720523	2.87E-30	7.62E-28	-0.3801048	2.46E-12
KRT17	0.680596081	0.59340715	-0.1977765	6.80E-30	1.81E-27	-0.4652854	1.96E-18
LSR	0.044625749	0.05635227	0.33659737	1.70E-29	4.52E-27	-0.3820452	1.86E-12
EVI2A	0.895181612	0.80832208	-0.1472501	1.74E-29	4.63E-27	-0.4652769	1.96E-18
VTCN1	0.567005944	0.6052883	0.09425861	8.11E-28	2.16E-25	-0.3929392	3.80E-13
CCDC8	0.339028487	0.45445323	0.42272532	8.37E-28	2.23E-25	-0.803562	5.89E-73
FAXDC2	0.205830006	0.27685114	0.42765713	9.47E-28	2.52E-25	-0.622874	1.87E-35
MAGEC2	0.814342286	0.75542977	-0.1083377	1.99E-27	5.29E-25	-0.4745676	3.29E-19
HIST3H2A	0.058873261	0.14345096	1.28487312	4.38E-27	1.17E-24	-0.4688145	1.00E-18
ZNF888	0.801174117	0.66737686	-0.2636142	5.26E-27	1.40E-24	-0.4333495	6.06E-16
CD3E	0.454622814	0.41513201	-0.1310999	5.90E-27	1.57E-24	-0.3762556	4.24E-12
AC083900.1	0.925400025	0.85611308	-0.1122758	2.67E-26	7.09E-24	-0.5849595	1.72E-30
MAGEB1	0.856104555	0.80280605	-0.0927355	4.19E-26	1.11E-23	-0.5323198	1.36E-24
FMO2	0.528755439	0.43607852	-0.2780127	6.66E-26	1.77E-23	-0.6173484	1.09E-34
XAGE5	0.765070006	0.7178789	-0.0918513	1.08E-24	2.88E-22	-0.546685	4.22E-26
KCNJ1	0.504788486	0.55347193	0.13283114	3.12E-24	8.30E-22	-0.3176319	7.32E-09
CABP4	0.801572271	0.77353492	-0.0513662	6.73E-24	1.79E-21	-0.3641633	2.24E-11
KRTCAP3	0.450190784	0.57529649	0.35376914	4.11E-23	1.09E-20	-0.765409	2.95E-62
CD5	0.792796658	0.73745254	-0.1044007	4.51E-23	1.20E-20	-0.4092001	3.15E-14
GOLGA6L2	0.766540574	0.7053604	-0.1200016	6.86E-23	1.83E-20	-0.4347328	4.79E-16
CDH16	0.360074716	0.4219852	0.22889609	2.21E-22	5.88E-20	-0.4795833	1.23E-19
MT1E	0.087419551	0.16813249	0.9435707	1.12E-21	2.99E-19	-0.4293932	1.18E-15
BTNL3	0.626661037	0.56574001	-0.1475461	2.17E-21	5.76E-19	-0.3201204	5.50E-09

HOXC10	0.124474008	0.10893422	-0.1923873	2.55E-21	6.78E-19	-0.4877101	2.40E-20
HSD17B14	0.39738273	0.3548458	-0.1633369	6.62E-20	1.76E-17	-0.466345	1.60E-18
AKR1B1	0.278232067	0.25447149	-0.1287847	1.51E-19	4.01E-17	-0.5099605	2.19E-22
LINC01151	0.717012448	0.59905523	-0.2593091	2.52E-19	6.69E-17	-0.4131207	1.70E-14
SMIM3	0.494222369	0.43473589	-0.1850211	2.74E-19	7.28E-17	-0.7653048	3.13E-62
PAGE1	0.51862802	0.47684973	-0.1211655	3.25E-19	8.64E-17	-0.3324871	1.28E-09
SLC27A2	0.688250978	0.57814568	-0.2515017	5.11E-19	1.36E-16	-0.4621691	3.52E-18
HIF1A-AS2	0.243095718	0.20760335	-0.2276947	1.75E-18	4.67E-16	-0.5126823	1.20E-22
FABP7	0.748029026	0.63923622	-0.2267451	1.87E-18	4.97E-16	-0.642258	2.90E-38
PGF	0.385625989	0.35230863	-0.1303625	4.32E-18	1.15E-15	-0.6407899	4.81E-38
SLC10A5	0.714909721	0.53244905	-0.4251176	7.85E-18	2.09E-15	-0.4021596	9.42E-14
PCGEM1	0.84849339	0.72234245	-0.2322205	1.05E-17	2.80E-15	-0.4481246	4.60E-17
FMR1NB	0.948374906	0.92294795	-0.0392082	1.23E-17	3.26E-15	-0.50132	1.41E-21
TMEM244	0.762389641	0.72912925	-0.0643539	1.67E-17	4.45E-15	-0.399729	1.37E-13
RNF149	0.807127239	0.74334434	-0.1187655	5.85E-16	1.56E-13	-0.323691	3.64E-09
RAB30-AS1	0.327695803	0.43133425	0.39644907	1.84E-15	4.89E-13	-0.3635884	2.42E-11
PAGE5	0.636053763	0.59637943	-0.0929182	2.35E-15	6.24E-13	-0.4251804	2.39E-15
PPP1R2P10	0.834209458	0.85903763	0.04231166	3.35E-15	8.91E-13	-0.5789435	9.19E-30
PON3	0.464661842	0.50724284	0.12649542	1.89E-14	5.04E-12	-0.5860266	1.27E-30
TRIP13	0.914136261	0.88719183	-0.0431631	2.09E-14	5.56E-12	-0.4647386	2.17E-18
CHRNA9	0.593840671	0.60854905	0.03529765	2.11E-14	5.62E-12	-0.3650278	2.00E-11
TM4SF18	0.241748804	0.20182593	-0.2603972	2.61E-14	6.95E-12	-0.5003301	1.74E-21
POF1B	0.638368148	0.55124687	-0.2116901	5.23E-14	1.39E-11	-0.6070526	2.66E-33
NUDT13	0.522746183	0.50086566	-0.0616869	7.73E-13	2.06E-10	-0.3652903	1.93E-11
IGFBP7	0.262771126	0.28006563	0.09195818	8.22E-13	2.19E-10	-0.3714096	8.34E-12
ZNF382	0.050981852	0.09845267	0.94944648	1.25E-12	3.32E-10	-0.6369586	1.78E-37
PAGE3	0.859308233	0.81069129	-0.0840231	1.64E-12	4.35E-10	-0.4383427	2.57E-16
ZNF280A	0.638455115	0.60506244	-0.0775012	2.54E-12	6.75E-10	-0.321413	4.74E-09
XAF1	0.366506141	0.31356702	-0.2250636	3.64E-12	9.68E-10	-0.4229309	3.47E-15
PAGE2B	0.821118355	0.79816797	-0.0408978	4.29E-12	1.14E-09	-0.5432453	9.84E-26
EPO	0.102589584	0.15394952	0.58557312	3.00E-11	7.97E-09	-0.3761688	4.30E-12
SERPINF1	0.513624633	0.48354763	-0.0870564	3.95E-11	1.05E-08	-0.6356401	2.78E-37
C1orf116	0.721599653	0.75439865	0.06412845	1.64E-10	4.36E-08	-0.5741619	3.40E-29
C2CD4D	0.166366915	0.13778191	-0.2719821	5.01E-10	1.33E-07	-0.4466112	6.02E-17
RGPD2	0.366810635	0.42322042	0.20637379	5.99E-10	1.59E-07	-0.3825508	1.73E-12
PLA2G12B	0.692466112	0.62901194	-0.1386561	6.23E-10	1.66E-07	-0.6655879	6.29E-42
CRYZ	0.117033679	0.1083081	-0.1117826	7.15E-10	1.90E-07	-0.4053394	5.77E-14
EVC2	0.474504845	0.47057643	-0.0119938	2.09E-09	5.57E-07	-0.6234331	1.56E-35
WWC2-AS2	0.621523776	0.69285663	0.15674726	3.51E-09	9.33E-07	-0.4252637	2.36E-15
MKRN3	0.755362489	0.71852506	-0.0721307	1.15E-08	3.06E-06	-0.667288	3.30E-42
LCN12	0.511422352	0.52734887	0.04424249	1.38E-08	3.67E-06	-0.3580199	5.09E-11
RASSF10	0.132241365	0.23365208	0.82118833	3.21E-08	8.54E-06	-0.3517212	1.16E-10
SPINT2	0.062509924	0.07039313	0.17134937	4.13E-08	1.10E-05	-0.5218761	1.53E-23
SSX9	0.862752114	0.82552908	-0.0636271	9.84E-08	2.62E-05	-0.3600977	3.87E-11
S100A4	0.55254661	0.57795495	0.06486088	1.98E-07	5.26E-05	-0.4583746	7.13E-18
TGM1	0.863030556	0.83690586	-0.0443463	2.68E-07	7.13E-05	-0.672783	3.99E-43
EHF	0.549980232	0.57778019	0.07114097	2.92E-07	7.77E-05	-0.3553059	7.28E-11
PAGE2	0.801822844	0.78779086	-0.0254708	9.12E-07	0.00024272	-0.3566912	6.07E-11
FAM153A	0.528660534	0.56134067	0.08653495	1.27E-06	0.00033694	-0.358532	4.76E-11
ODAM	0.731003739	0.73652869	0.01086294	1.34E-06	0.00035532	-0.4156576	1.13E-14
CLEC18C	0.541279483	0.52510716	-0.0437618	2.73E-06	0.00072626	-0.4253278	2.33E-15
FOXI1	0.670659787	0.68620116	0.03305047	7.67E-06	0.00204069	-0.3499356	1.46E-10
CRYAB	0.229524347	0.21736462	-0.0785301	8.11E-06	0.00215801	-0.3705741	9.36E-12
NUDT12	0.244337703	0.24064523	-0.0219686	1.74E-05	0.0046375	-0.4910526	1.21E-20
DCAF12L1	0.434590218	0.49032443	0.17408093	6.41E-05	0.01705343	-0.6366119	2.00E-37
DEFB1	0.280649979	0.2940125	0.06710553	8.74E-05	0.02323598	-0.3406426	4.72E-10
XIST	0.833775652	0.81145544	-0.0391474	9.26E-05	0.02463176	-0.8747183	4.12E-101
HOXA10-AS	0.134208187	0.16036768	0.25691076	0.00011276	0.02999306	-0.5824708	3.45E-30
ABCA6	0.491783527	0.44627512	-0.14009	0.00016563	0.04405691	-0.3464768	2.27E-10
AKR7A3	0.205926668	0.22611196	0.13490665	0.00018428	0.04901723	-0.3647446	2.07E-11
